# Selection on non-social traits limits the invasion of social cheats

**DOI:** 10.1111/j.1461-0248.2012.01805.x

**Published:** 2012-05-29

**Authors:** Andrew D Morgan, Benjamin J Z Quigley, Sam P Brown, Angus Buckling

**Affiliations:** 1 Department of Zoology, University of OxfordSouth Parks Road, Oxford, OX1 3PS, UK; 2 Institute of Evolutionary Biology, School of Biological Sciences, University of EdinburghKing's Buildings, Edinburgh, EH9 3JT, UK; 3 Biosciences, University of ExeterPenryn, Cornwall, TR10 9EX, UK

**Keywords:** Bacteria, cheat, coevolution, phage, positive frequency dependence, spatial structuring

## Abstract

While the conditions that favour the maintenance of cooperation have been extensively investigated, the significance of non-social selection pressures on social behaviours has received little attention. In the absence of non-social selection pressures, patches of cooperators are vulnerable to invasion by cheats. However, we show both theoretically, and experimentally with the bacterium *Pseudomonas fluorescens*, that cheats may be unable to invade patches of cooperators under strong non-social selection (both a novel abiotic environment and to a lesser extent, the presence of a virulent parasite). This is because beneficial mutations are most likely to arise in the numerically dominant cooperator population. Given the ubiquity of novel selection pressures on microbes, these results may help to explain why cooperation is the norm in natural populations of microbes.

## Introduction

Spatial structure can favour the maintenance of indiscriminate cooperation through increases in the genetic similarity of interacting individuals, but cooperating patches can still be invaded by ‘cheats’ that benefit from the cooperative behaviour, but do not pay the costs of participating in it (Hamilton 16, 17; Nowak 36). However, where explicitly investigated, cooperation is the norm in natural populations of microbes. For example, only eight of 345 natural isolates of *Pseudomonas aeruginosa* were found to be cheats with respect to the production of extracellular iron-scavenging siderophores (Bodilis *et al*. 3). While this could be explained to some extent by assuming cheats rarely encounter patches of cooperators through immigration, a number of recent studies demonstrate how easily cheats can be generated by mutation, and subsequently increase in frequency (Rainey & Rainey 39; Harrison & Buckling 18; Sandoz *et al*. 43; Kohler *et al*. 24; Racey *et al*. 38). Cheat invasion can be prevented if a single locus that confers cheating behaviour has a negative effect on another fitness trait (Foster *et al*. 13), but such pleiotropy does not seem to be common in microbial cooperative behaviours (West *et al*. 50), and certainly does not limit the invasion of siderophore cheats in laboratory adapted strains of *Pseudomonas aeruginosa* (Harrison & Buckling 18). Here, we investigate a simple and potentially ubiquitous mechanism limiting cheat invasion in natural populations of microbes: strong non-social selection pressures.

Environmental change, particularly changes in other interacting organisms such as predators (Feldman *et al*. 11), parasites (Koskella & Lively 26), and competitors (Joshi & Thompson 21), can result in maladaptation of populations to their local environment, and hence strong selection (Bell 2). Assuming new patches are founded by cooperating cells, as patches of cooperators are more productive and so produce more migrants (Hamilton 16, 17; Nowak 36), beneficial mutations are more likely to arise by chance in the numerically dominant cooperator population, than in rare invading cheats. If these non-social beneficial mutations are under stronger selection than the cheating mutation, cooperative alleles will hitchhike with the beneficial mutations and the cheating mutation will decrease in frequency by clonal interference (Fisher 12; Schiffels *et al*. 45) from the beneficial non-social mutation. Crucially, there are likely to be many selective sweeps due to continual environmental change (especially with a coevolving parasite), or if multiple mutations are required to reach fitness optima. Hence, although a cheat may gain a beneficial non-social mutation, or a cheat may emerge from a cooperator population that has gained one beneficial non-social mutation, additional beneficial mutations are more likely to arise in the numerically dominant cooperator population.

To test our hypothesis of indirect positive frequency-dependent cooperator fitness when adapting to novel environments, we developed a theoretical model and tested it by evolving populations of the bacterium *Pseudomonas fluorescens* SBW25. Wild-type *P. fluorescens* produces extracellular siderophores that scavenge iron from the environment and are re-adsorbed by cells (Cornelis 9), and we establish here that siderophore production is a cooperative trait, as is the case in the closely related bacterium, *Pseudomonas aeruginosa* (Griffin *et al*. 15). We then evolved mixed populations of wild-type *P. fluorescens* and an isogenic mutant cheat strain at various starting frequencies. Strong directional selection was created by culturing them for *c*. 40 generations in iron-limited nutrient media, a novel environment for these non-laboratory adapted bacteria. Moreover, some populations were coevolved with a virus (the lytic DNA bacteriophage, SBW25Φ2), resulting in continual strong selection for resistance (Buckling & Rainey 7; Morgan *et al*. 34). Note that competition occurs only within-, and not between-, patches (i.e. soft selection; (Wallace 49)) in both our model and experiments, although we implicitly assume the operation of global regulation and hence between-patch competition (i.e. hard selection) to explain why cooperators are likely to be the first to colonise empty patches.

## Materials and methods

### Theoretical methods

To explore the consequences of ongoing non-social selection pressures on the evolution of cooperation, we constructed a simple model to calculate the expected change Δ*p* in the frequency *p* of a cooperative allele across a period of environmental adaptation (incorporating both selection and subsequent population recovery to a static carrying capacity *K*). We assumed that selection on the social trait is weak, and that selection is strong on an uncorrelated trait that drives environmental adaptation, such as resistance to a parasite. Together these assumptions allowed the calculation of Δ*p* as the probability weighted sum of changes when cooperators alone gain resistance (Δ*p* = 1 − *p*; cooperators fix); when cheats alone gain resistance (Δ*p* = −*p*; cheats fix); and when neither or both cooperators or cheats gain resistance (Δ*p* = 0). Assuming a binomial distribution of resistance mutations, and using a framework based on the Price Equation (Gardner 14), we were able to calculate predicted changes in *p* (see Figure S1).

### Experimental materials and methods

#### Microcosms

Populations of *P. fluorescens* were grown in static microcosms, which consisted of 30 mL glass universals containing 6 mL of King's media B (KB). We used static microcosms for consistency with previous studies (Griffin *et al*. 15; Kummerli *et al*. 28), where siderophore cheats can readily invade, presumably because of the high diffusibility of siderophores in liquid media. Hence each microcosm behaved as a single mixed patch with no migration, and so the role of kin selection was considered to be negligible. To create iron limited conditions, 70 μg mL^−1^ of apotransferrin was added to chelate the iron, and sodium bicarbonate (which is needed to raise the pH for the chelating activity of the apotransferrin) was added to a final concentration of 20 mM (Griffin *et al*. 15). Additionally the microcosms were supplemented with an excess of pantothenic acid (0.0024%) (Rainey & Travisano 40) to allow the cost-free growth of the marked wild-type (panB) strain (see below).

#### Determining fitness of cheat in presence and absence of cooperator

We first determined whether the cheat strain behaved as cheat by having higher fitness in the presence of the wild-type cooperator, than it was alone. Six replicate microcosms were set up per treatment. The treatments were: 100% (monoculture) cheat, 100% (monoculture) wild-type, 50%: 50% wild-type to cheat ratio, and 99.99%: 0.01% wild-type to cheat ratio. These ratios were chosen as they were the highest and lowest starting cheat frequencies used in the subsequent evolution experiment, allowing us to determine whether the cheat could invade across the whole range of frequencies in the absence of evolution. We note that by examining these extreme frequencies we assume a monotonic frequency effect. The wild-type bacterium was an isogenic *Pseudomonas fluorescens* SBW25 marked with a deletion of the entire *panB* gene, turning it into a pantothenate auxotroph (Rainey & Travisano 40). This marker has no negative effects on fitness when grown in an excess of pantothenate (Rainey & Travisano 40), but allows the strain to be distinguished from the cheat on agar plates (see the ‘enumeration of wild-type and cheat’ section below). The cheat bacterium was an isogenic strain of *P. fluorescens* SBW25 that had the *pvdL* gene knocked out (ΔpvdL) (Moon *et al*. 33): *pvdL* is predicted to encode a non-ribosomal peptide synthase involved in pyoverdine chromophore biosynthesis (Moon *et al*. 33). Approximately 10^7^ cells in the correct ratios were added to each microcosm. The precise starting ratio was determined by serial plating as described in the ‘ enumeration of wild-type and cheat’ section below. The microcosms were incubated at 28 °C, static, with loose caps for 48 hours. The final numbers of the wild-type and cheat were also determined by serial plating. Fitness of the cheat was calculated using the estimated Malthusian parameters (*m*), where *m* = ln(*N*_f_/*N*_0_) where *N*_0_ is the starting density and *N*_f_ is the final density (Lenski *et al*. 29).

To determine whether the cheat increased in frequency when mixed with the wild-type, we calculated *v*, which is the relative change in frequency of the cheat over time relative to the wild-type; where *v* = *x*_2_(1 − *x*_1_)/*x*_1_(1 − *x*_2_), *x*_1_ = starting proportion of cheat, and *x*_2_ = final proportion of cheat (Ross-Gillespie *et al*. 41). Therefore *v* was greater than 1 if the cheats increased as a proportion of the population, and less than 1 if they declined in proportion. Note that we used *v* here, rather than relative Malthusian parameters of competitors (Lenski *et al*. 29), for consistency with the subsequent evolution experiment. Relative Malthusian parameters, which estimate relative exponential growth rates, are inappropriate over the multiple cycles of exponential and stationary phase growth experienced during the evolution experiment.

#### Evolving populations

Twelve replicate microcosms were set up for each ratio of wild-type (WT) to cheat bacteria tested (100% WT, 100% cheat, 50% WT: 50% cheat, 90% WT: 10% cheat, 99% WT: 1% cheat, 99.9% WT: 0.1% cheat, and 99.99% WT: 0.01% cheat). The WT and cheat were mixed at the specified ratio, and *c*. 10^7^ of the mixed bacterial cells were added to each microcosm. Approximately 10^5^ particles of phage SBW25Φ2 (Buckling & Rainey 7) were added to half of the replicates of each of the different ratios. The microcosms were incubated at 28 °C, static, with loose caps.

Every 48 h, 1% of the cultures were transferred to fresh microcosms. Every second transfer, portions of the cultures were frozen in 20% glycerol and stored at −86 °C. The experiment continued for a total of 6 experimental transfers which allowed time for selective sweeps as the bacteria adapted to the novel abiotic (novel media) and biotic (coevolving bacteriophage) environments. The ratios of the wild-type to the cheat were determined by serial plating at the start of the experiment and at transfer 6 (at the end of the experiment), as described in the ‘enumeration of wild-type and cheat’ section below.

To calculate whether the cheat increased or decreased in frequency we used *v*, as described in the section above, and the data was log_10_ transformed to meet assumptions of normality. Therefore, when the data was log_10_ transformed, *v* was greater than 0 if the cheats increased as a proportion of the population, and less than 0 if they declined in proportion. The data were analysed with a General Linear Model, fitting presence or absence of phage as a factor, the starting proportion of cheat as a covariate, an interaction term between them, and a quadratic term.

#### Enumeration of wild-type and cheat

The wild-type and cheat were enumerated by serial dilution and plating on to King's agar B, and vitamin-free casein amino acid agar [KB agar with 20 g proteose peptone no. 3 per litre replaced with 10 g vitamin-free casamino acids (Buckling *et al*. 8)]. The wild-type, due to the knock out of its *panB* gene, formed small or no colonies on the vitamin-free casein amino acid agar, while the cheat formed normal sized colonies. The cheat colonies were counted and the density determined was subtracted from the total (wild-type + cheat) bacteria density determined by the counts on the KB agar to give the density of the wild-type.

## Results

### Theoretical results

**Figure 1:**
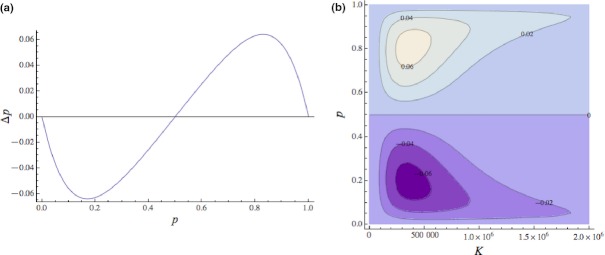
Strong selection creates frequency and density dependence on a social trait. The expected change in cooperative allele frequency Δ*p* is plotted as a function of: (a) the frequency of cooperation *p*, and (b) the frequency of cooperation *p* and carrying capacity *K*. The social trait experiences positive frequency dependence, with Δ*p* positive when *p* > 0.5. The frequency-dependent effect is maximised for intermediate densities *K* as these regions are where the bias in mutational supply are greatest. The model is derived in Figure S1. The per-cell probability of beneficial non-social mutation is *q* = 1 × 10^−5^, and density (a) is *K* = 5 × 10^5^.

The resulting model (derived from the Price equation, see the Supporting information) predicted indirect positive frequency-dependent selection on the cooperative trait, as dominant cooperators (high *p*) are more likely to gain mutations that are favourable in the face of strong non-social selection (Fig. 1a). The indirect positive frequency dependence is itself density dependent (maximal for intermediate densities *K*), due to the maximisation of the bias in mutational supply at intermediate densities (Fig. 1b and Figure S1).

Simulation results (Figure S3) which explicitly include the costs and benefits of cooperation, confirm the model predictions, demonstrating that these results hold when these further complexities and others, such as explicit host-parasite dynamics are added. The model we present in this paper thus provides us with a simple and robust framework that arrives at analytically tractable results. Moreover, it reveals a biologically plausible mechanism by which the maintenance of cooperation may be facilitated in natural populations.

### Experimental results

We then tested our predictions with populations of *P. fluorescens*. Firstly, we established that the cheat strain of *P. fluorescens* SBW25 [a *pvdL* knock-out that did not produce the primary siderophore, pyoverdin (Moon *et al*. 33)] had a selective advantage over the pyoverdin-producing wild-type cooperator in mixed populations, when non-social selection pressures were relatively unimportant, that is, that it behaved as a cheat. We therefore carried out assays for the two strains over time periods too short (48 h, *c*. six generations) for beneficial non-social mutations to spontaneously arise and sweep to high frequencies. Fitness of the cheat was significantly greater when it was mixed with the wild-type than as a monoculture at both 50% cheat: 50% wild-type ratios (*T* = 2.67, *P* = 0.032, Figure S6a); and 99.99% wild-type: 0.01% cheat ratios (*T* = 3.21*, P* = 0.018, Figure S6a). Note that previous work has established a large growth cost of the cheat relative to the wild-type when grown as monocultures in iron-limited media (Moon *et al*. 33). There was a cost to cooperation, as the fitness of the wild-type was significantly lower when it was mixed with the cheat at 50% cheat: 50% wild-type starting ratio than as a monoculture (*T* = 3.52, *P* = 0.008, Figure S6b). However, when the wild-type was mixed with the cheat at starting ratios of 99.99% wild-type: 0.01% cheat, the fitness of the wild-type was not significantly different to its fitness in monoculture (*T* = 1.3 *P* = 0.229, Figure S6b), presumably because the cooperators were at such high frequency, there were plenty of siderophores available, and so their growth wasn't diminished.

**Figure 2:**
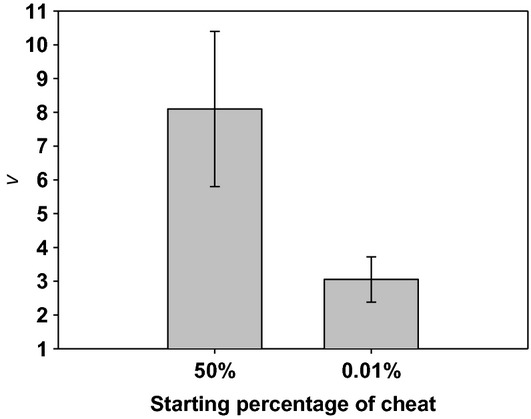
Invasion of cheat over a period too short (48 h) for evolution to occur, at high (50%) and low (0.01%) starting frequencies of cheat. *v* is a measure of invasion of the cheat where *v* > 1 indicates an increase in the frequency of the cheat (invasion) as a proportion of the population, whereas *v* < 1 indicates that the cheat has declined in proportion. Error bars are ± 1 standard error of the mean.

We also calculated *v*, which is a measure of the invasion of the cheat: where if *v* > 1, then the cheat had increased in frequency as a proportion of the population; and if *v* < 1, then the cheat had declined in proportion. The cheat had increased from its starting frequency, as *v* was significantly greater than 1, in both high (50%) starting frequencies of cheat (*T* = 3.09, *P* = 0.027, Fig. 2) and low (0.01%) starting frequencies of cheat (*T* = 3.06, *P* = 0.028, Fig. 2).

To determine whether strong non-social selection pressures could maintain cooperation, we mixed cheats at starting frequencies from 0.1% to 50% with the wild-type, and also set up monocultures of wild-type and cheat. These were cultured over a period of 40 generations, where significant adaptation to the abiotic environment (the novel media) could occur, and half of these populations were exposed to a continual strong biotic selection pressure in the form of a coevolving phage. Adaptation to the abiotic environment was demonstrated by higher monoculture densities of both cheats and cooperators in the absence of phage at transfer 6 vs. transfer 2 (paired t-tests, wild-type *T* = 7.04, *P* = 0.001, cheat *T* = 3.3, *P* = 0.021). Adaptation to the biotic environment was shown over this timescale by cycles of coevolution with phages in monocultures of both wild-type and cheat, where phage infectivity (virulence) and bacterial resistance increases through time, as shown in Figure S7, consistent with previous studies (Buckling & Rainey 7; Brockhurst *et al*. 4). The rate of coevolution with phage did not differ significantly between the wild-type and the cheat, as their negative slopes of resistance to phages through time were not significantly different: (*T* = 0.14, *P* = 0.892, Figure S7), neither did mean resistance to contemporary phages (*T* = 1.63, *P* = 0.155, Figure S7), which suggests that there are no pleiotropic effects of knocking out the *pvdL* gene in the cheat, or the *panB* gene in the cooperator, on their ability to evolve resistance to phage. Recent Whole Genome Sequencing has confirmed that there is a mutational, rather than physiological basis, to phage resistance (Pauline Scanlan, personal communication).

**Figure 3:**
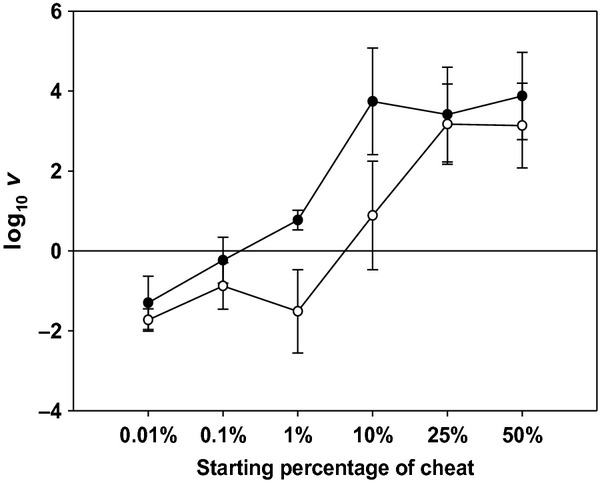
The change in frequency of cheats in the presence (white circles) and absence (black circles) of phages at different starting frequencies of cheats. Log_10_ *v* is a measure of the invasion of the cheat where a positive value indicates an increase in the frequency of the cheat (invasion) as a proportion, whereas negative values indicate that the cheat has declined in proportion. Error bars are ± 1 standard error of the mean.

As hypothesized from our model results, the fitness of cheats (and cooperators) was positive frequency dependent (linear *F*_1,66_ = 38.42, *P* < 0.001, and quadratic term *F*_1,66_ = 12.09, *P* = 0.001 Fig.3), as they evolved to the abiotic environment, such that cheats declined in frequency at low initial frequencies and increased at high initial frequencies. In the absence of phages, cheats declined in frequency when they started at a frequency of 0.1% or less (Fig. 3). We note that because the data in Fig. 3 was log_10_ transformed, then if *v* > 0, the cheats had increased as a proportion of the population, and if *v* < 0, then they declined in proportion. The departure from the theoretical threshold of 50% (Fig. 1 and Figure S3) follows from non-zero costs of cooperation generally favouring cheats. As the fitness of both cheat and cooperators was positive frequency dependent, it suggests that there were no or limited pleiotropic effects of knocking out the *pvdL* gene in the cheat or the *panB* gene in the cooperator.

The presence of phages caused a significant net reduction in cheat fitness (*F*_1,66_ = 4.84, *P* = 0.031, Fig. 3) (stronger non-social selection) because at all starting frequencies (apart from 50%) the cheat was the minority, hence the wild-type population acquired resistance mutations more rapidly. An alternative, but not mutually exclusive, explanation is that the cheat is intrinsically less able to evolve resistance than the cooperator, but this seems unlikely (as discussed above), as rates of coevolution and contemporary resistance to phages was significantly different between monocultures of cheat and phage (Figure S7). We were surprised that there was no significant interaction (*F*_1,66_ = 0.24, *P* = 0.624) between the presence of phages and starting frequency of cheats: we assumed phages would reduce cheat fitness more at low frequencies. The absence of a frequency-dependent effect of phage most likely reflects the stronger selective effect of the abiotic environment compared with phage.

## Discussion

Our results show that selective sweeps in response to strong selection can indirectly prevent the invasion of single patches of cooperators by cheats. Moreover, as a result of this mechanism, fitness of both cheats and cooperators is indirectly positive frequency dependent, whereas models that ignore non-social selection typically either show frequency independence or negative frequency dependence, depending on population structure and other biological details (MacLean & Gudelj 30; Ross-Gillespie *et al*. 42). This indirect positive frequency dependence results from the fitness benefits conferred by the non-social mutations being significantly greater than the fitness benefit of the social trait (clonal interference; Fisher 12), and the numerically dominant genotype having the greater probability of acquiring beneficial non-social mutations (genetic hitch-hiking).

This mechanism could of course also contribute to preventing the invasion of cheats by rare cooperators, as well as vice versa, so this mechanism can only provide a net advantage to indiscriminate cooperation when cooperation is already the dominant strategy within the population as a whole. Such an advantage to indiscriminate cooperation can readily arise in a spatially structured populations (i.e. a metapopulation) under hard selection where kin competition is relaxed [e.g. if local populations can expand (Taylor 47)]. Cheats can appear in patches of cooperators via immigration (from patches where cheats are present) or, particularly in the case of microbes, by mutation (Rainey & Rainey 39; Harrison & Buckling 18; Sandoz *et al*. 43; Kohler *et al*. 24; Racey *et al*. 38). Under these circumstances, but in the absence of non-social selection pressures, cheats are likely to have a fitness advantage when rare, as there is a high probability of encountering patches of co-operators to exploit (Ross-Gillespie *et al*. 41). Strong non-social selection pressures could then prevent their invasion. While cheats could of course continually mutate from cooperators that are well adapted to current environmental conditions, cheat invasion would be similarly prevented by further environmental change, such as a coevolving parasite.

We have only considered here the impact of positive selection, resulting in selective sweeps of beneficial mutations, on the evolution of cooperation. Selection imposed by the physical environment or natural enemies may also be diversifying, resulting in negative frequency-dependent fitness (Koskella & Lively 27), which may promote coexistence of social strategies as a result of genetic linkage between the social trait and the trait experiencing diversifying selection. Indeed, a recent theoretical study demonstrated the importance of hitch-hiking with non-social alleles for the invasion of cooperation alleles (and coexistence with selfish alleles) in metapopulations, under the assumption that genetically diverse groups are more productive (social heterosis) (Santos & Szathmary 44). Moreover, demographic changes resulting from strong selection may also have unexpected effects on cooperation. For example, population crashes may further decrease the probability of rare cheat invasion, but may also reduce cooperator fitness if threshold densities are required for the benefits of cooperation to be realised (Kadam & Velicer 22; Brockhurst *et al*. 5).

Whilst these results have little relevance to frequently recombining populations, where genetic hitch-hiking is likely to be short-lived (Barton 1), selective sweeps may play a significant role on the evolution of social traits for many, largely asexual, microbial populations (including those used in experimental evolution studies that have not been pre-adapted to laboratory conditions). Microbes experience strong selection in many natural environments as a result of natural enemies, for example, competitors (Vos & Velicer 48), microbial predators (Morgan *et al*. 35), and environmental pollution (McArthur & Tuckfield 32), and cheats can be readily generated by loss of function mutations (Rainey & Rainey 39; Harrison & Buckling 18; Sandoz *et al*. 43; Kohler *et al*. 24; Racey *et al*. 38). The results are particularly relevant to pathogenic bacteria, which regularly encounter strong selection pressures in the form of antibiotics (Davies & Davies 10; MacLean *et al*. 31), immune pressure (Suerbaum & Josenhans 46), and phages (O'Flynn *et al*. 37); and whose virulence, infection duration, and transmissibility are affected by the production of public goods, such as siderophores (Harrison *et al*. 19), proteases (Kennan *et al*. 23), and biofilm polymer (Hoffmann *et al*. 20). Indeed, our results may help to explain why *P. aeruginosa* cheats reached much higher frequencies in acute infections that were not treated with antibiotics compared with those that were (Kohler *et al*. 24, 25). Finally, the study offers a cautionary note to the potential therapeutic gains from introducing engineered cheat lineages into recalcitrant infective populations (Harrison *et al*. 19; Brown *et al*. 6): despite their social advantages, introduced cheats face a potentially formidable frequency-dependent barrier to invasion in the context of non-social selection.

## SUPPORTING INFORMATION

As a service to our authors and readers, this journal provides supporting information supplied by the authors. Such materials are peer-reviewed and may be re-organized for online delivery, but are not copy-edited or typeset. Technical support issues arising from supporting information (other than missing files) should be addressed to the authors.
