# Supplementary material for: Selection on non-social traits limits the invasion of social cheats
Source: Ecol Lett. 2012 May 29;15(8):841–6. doi: 10.1111/j.1461-0248.2012.01805.x (PMC3444687; doi:10.1111/j.1461-0248.2012.01805.x)
Supplement: Supplementary file 1 [file ele0015-0841-SD1.doc]

**Supporting Information**

**1. Theoretical results**

**Life cycle**

Consider a haploid population at carrying capacity, *K*, with a proportion *p* of the individuals carrying a cooperative allele. The population is then put under strong selection by some environmental change, forcing a severe bottleneck. The remaining individuals are those that have the ability to persist in light of abiotic (novel environment) and/or biotic (phage) selection pressures. Such resistant individuals act as founders, as the population then experiences re-growth and recovers back to the original carrying capacity.

To calculate whether the proportion of cooperators *p* is increasing or decreasing across a life-cycle, we calculate *∆p* = *p' - p,* where *p'* is the proportion of cooperators following bottlenecking and regrowth to carrying capacity. Given that

*w_i_* = fitness of individual *i* (number of descendent offspring of *i* following selective bottleneck and regrowth) and *p_i_* = frequency of cooperator allele (0 or 1) in individual *i*, the change in the frequency of cooperators can be captured in the selection term of the Price equation :

$=E(p_{i}w_{i})-E(p_{i})E(w_{i})$ [1]

Given that the expected value of individual cooperator frequency is just the population frequency, *p*, and that the expected value of individual fitness is 1 (as the population is not expanding across the lifecycle), then the above equation becomes:

$$\Delta p=E(p_{i}w_{i})-p$$

$=\frac{\sum_{i=1}^{N} p_{i}w_{i}}{N}-p$ [2]

Given that *p*_i_=1 for cooperators and *p*_i_=0 for cheaters and that on average we have *pN* cooperators and (1-*p*)*N* cheaters, equation 2 becomes:

$=\frac{\sum_{i=1}^{pN} w_{c}}{N}-p$ [3]

Note that the expected fitness of a cooperator is $\frac{\sum_{i=1}^{pN} w_{c}}{pN}$, therefore we have:

[4]

**Calculating Expected Fitness of A Cooperator**

To make use of equation [4] we need to calculate E[*w_c_*] under strong selection on an asocial trait. The expected fitness of a cooperator E[*w_c_*], must satisfy the identity *p'* = *p* E[*w_c_*], therefore we can calculate E[*w_c_*], as a function of who (cooperators and/or cheaters) gains resistance. C refers to cooperators and D to cheaters. If both lineages gain resistance then the frequency of cooperators will be on average unchanging (in the limit of weak selection on cooperation), i.e. *p'* = *p*, implying that E[*w_c_* | both C and D gain resistance] = 1. Similarly, if neither gain resistance then again *p'* = *p*, and therefore E[*w_c_* | neither C or D gain resistance] = 1. If cooperators alone gain resistance then we expect a selective sweep sending *p'* = 1, implying that E[*w_c_* | only C and not D gain resistance] = 1/*p.* Finally, if cheaters alone gain resistance we have a sweep in the opposite direction resulting in *p'* = 0 and E[*w_c_* ] only D and not C gain resistance] = 0. To calculate E[*w_c_*] we need to build a weighted sum of the above conditional expectations,

E[w_c_] = 1 (prob both cooperators and cheaters gain resistance + prob neither gain resistance) + 1/*p* (prob only cooperators gain resistance) [5]

Given a probability$\mu$ that a single bacterium gains resistance and a binomial distribution of resistant mutants, the probability of getting *no* resistant cooperators is simply$(1-\mu)^{pK}$. Therefore the probability of getting *any* resistant cooperators is $1-(1-\mu)^{pK}$. Similarly, the probability of getting no resistant cheaters is $(1-\mu)^{(1-p)K}$ and the probability of getting any resistant cheaters is $1-(1-\mu)^{(1-p)K}$. These probabilities allow the calculation of:

Prob. both C and D lineages gain resistance **= ($1-(1-\mu)^{pK}$) ($1-(1-\mu)^{(1-p)K}$) [6]**

Prob. neither lineage gain resistance **= ($(1-\mu)^{pK}$ ) ($(1-\mu)^{(1-p)K}$) [7]**

Prob. only C lineage gains resistance = ($1-(1-\mu)^{pK}$) ($(1-\mu)^{(1-p)K}$ [8]

The expected fitness of a cooperator is therefore (by substitution of Eq 6, 7 & 8 into Eq 5 and simplifying):

$E[w_{c}]=1+\frac{(2p-1)(1-\mu)^{K}}{p}-(1-\mu)^{Kp}-\frac{(p-1)(1-\mu)^{K-Kp}}{p}$ [9]

Substituting Eq 9 into Eq 4 and simplifying gives an expression for the change in a cooperative allele as a function of cooperative allele frequency (*p*) and demographic and mutational parameters (*K* and *µ*).

$\Delta p=(2p-1)(1-\mu)^{K}-p(1-\mu)^{Kp}-(p-1)(1-\mu)^{K-Kp}$ [10]

Plotting equation [10] as a function of cooperator frequency *p* we see that cooperators have a fitness advantage when more common (in the neutral limit when *p >* 0.5, figure 1a) as the more common lineage is more likely to acquire the resistance allele and then fix in the population. The model is symmetrical i.e. either cooperator or cheating lineages can gain a fitness benefit via this mechanism, though we focus on cooperators because we argue (in the main manuscript) that populations are initially likely to be dominated by cooperators.

As the initial population density increases (larger *K*) so does the likelihood that both lineages will acquire the resistance mutant, so preventing either from fixing and thus weakening the positive frequency dependent effect (figure 1b). Similarly, the effect weakens at low densities, as the likelihood increases that neither lineage will acquire the resistance trait (figure 1b).

**References**

1. Gardner A (2008) The Price equation. *Curr. Biol.* 18(5):R198-R202.

**2. Simulation results**

**Simulation results**

In order to explore our analytical predictions under broader scenarios, we developed some deterministic simulations. The simulations were developed in the context of a bacterial host, and an associated viral parasite i.e. biotic selection pressure. A strong abiotic selection pressure that acts to purge genetic diversity has an analogous effect as the strong biotic selection pressure (data not shown), however we focus on the interesting interaction between bacteria and phage in these explicit simulations.

Assume there are two types of host, *A* and *B*. Initially, the population contains entirely sensitive *A* and *B* individuals (denoted by the subscripts *A_S_* and *B_S_* respectively). Both *A_S_* and *B_S_* hosts are equally likely to evolve resistance to a parasite, *V*. Hosts that evolve resistance (at rate *μ*), are labeled *A_R_* and *B_R_*. The parasite can infect (and hence kill) both *A_S_* and *B_S_* hosts indiscriminately, but cannot infect resistant hosts.

Their dynamics are described by the following sets of differential equations:

where *r*= growth rate, *N*= total population number (i.e. *A_S_* + *B_S_* + *A_R_* + *B_R_*), *e*= scaling parameter, *Y*= ‘burst size’ of viral parasite (i.e. number of progeny released), *φ* = natural death rate of viral propagules. To allow for lineage extinctions, an arbitrary threshold of 1 was imposed to capture extinction events; lineages that fell below 1 became extinct.

In our simulations we are particularly concerned with how the initial conditions dictate the fate of competition between the A and B types. Figure S1 maps the outcome of the simulations over a range of initial type *A* frequencies (*p*) and total densities (carrying capacity, *K*). The simulations always begin with susceptible hosts, from which resistant mutants can emerge. The surface of the figure is made from the results of 10,000 simulations, ran over sufficient arbitrary time units such that consistent end points were reached. Analogous with the analytical results, there are four possible outcomes; A alone gain resistance (e.g. ii), B alone gain resistance (e.g. iv), and neither (e.g. i) or both gain resistance (e.g. iii). These outcomes are shown in figure S1, with explicit examples of typical simulation dynamics for each scenario detailed below in figure S2.


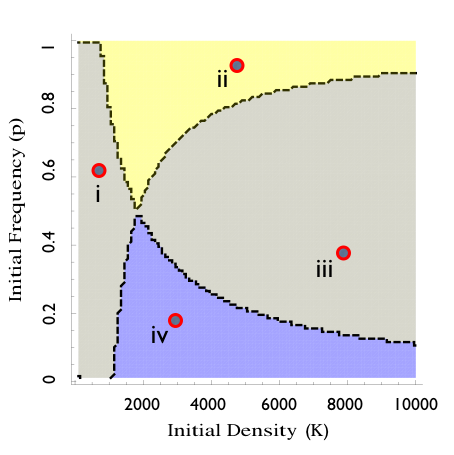


Figure S1: Phase plane diagram showing the regions type A and type B hosts are favored over a range of initial starting frequencies and densities. The top region shows where only A_R_ were present at the end of the simulation (e.g. ii). The bottom region shows where only B_R_ were present at the end of the simulation (e.g. iv). The left region shows where both A_S_ and B_S_ were present at the end of the simulations (e.g. i), and the right region shows where both A_R_ and B_R_ were present (e.g. iii). Representative time-series plots for each of the scenarios i-iv are pictured in figure S2. Parameters: *r* = 1, *μ* = 0.01, *y* = 10, *e* = 0.0001, *φ* = 0.2.

Figure S2: Examples of population dynamics from specified points in figure S1. Within each panel, the top graph is a time series showing numbers of *A_s_* (green, dashed), *B_s_* (red, dashed), *A_R_* (dark green), *B_R_* (dark red), and virus (purple) through arbitrary units of time. Note: viral numbers are scaled down (/15) such that the results can be easily presented on the same axes. The bottom graphs in each panel correspond to those above, showing the total proportion of A (*A_S_* + *A_R_*, green) and B (*B_S_* + *B_R_,* red) in the population. i) Neither A or B gain resistance; ii) A only gain resistance; iii) Both A and B gain resistance; iv) Only B gain resistance. Parameters: *r* = 1, *μ* = 0.01, *y* = 10, *e* = 0.0001, *φ* = 0.2.

These simulation results assume that there is no differential selection on A and B hosts i.e. the model is neutral with respect to host type. Under this assumption, the model demonstrates the positive frequency and density dependent advantage of any traits that find themselves associated with a resistance mutation. Associated genes will hitchhike to fixation, whilst all other genetic diversity is purged.

Adding explicit cooperation

The experimental results in the main manuscript are derived in the context of cooperators and cheats. Our neutral model can be very easily adapted to represent this scenario. From now on, imagine that type A hosts are cooperators (C) and type B hosts are cheats (D). If we introduce explicit costs *x* and benefits *b* of cooperation, the differential equations that represent the dynamics become:

Where *C_i_* = *C_S_*+ *C_R_*.

Holding fixed the benefit of cooperation (*b*=0.5), we can investigate what happens as we increase the cost of cooperation. Figure S1 can be re-drawn to include an increasing cost of cooperation. When there is no cost of cooperation, the outcome represented in figure S1 is recovered (see S3 A). With the model no longer being neutral, the frequency threshold above which we only find cooperators is shifted further above 0.5 as the cost of cooperation increases.


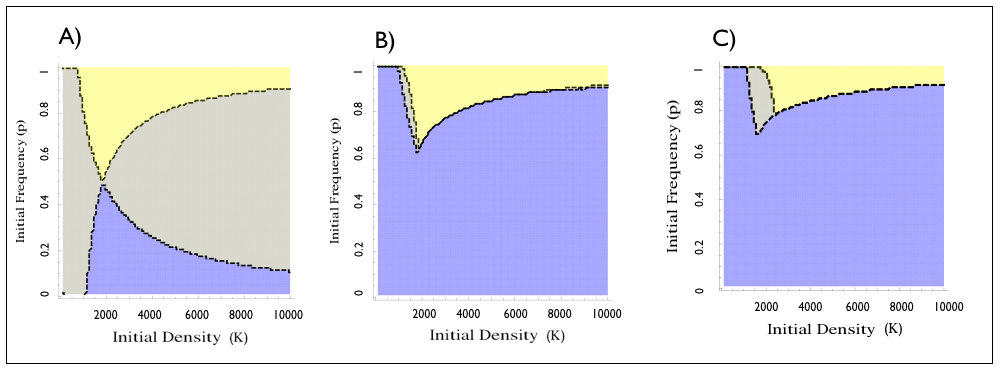


Figure S3: Re-drawing figure S1 with an increasing cost of cooperation. A) when there is no cost of cooperation (recovering figure S1). B) when there is a medium cost of cooperation, *x*=0.3. C) when there is a high cost of cooperation *x*=0.49. The area shaded blue represents the parameter space where cheats have reached fixation (only *D_S_* and/or *D_R_* present at the end of the simulation, no *C_S_* or *C_R_*). The area shaded yellow is where only cooperators are present (*C_R_*), and the area shaded grey demonstrates where *D_S_* are coexisting with *C_R_*.

We can see from figure S3 that as a cost of cooperation is introduced, cheats reach fixation where we would previously expect coexistence of neutral strains. This effect becomes stronger as the cost of cooperation increases. Given this cost, there are now four possible outcomes: neither cooperators or cheats gain resistance (e.g. v), coexistence of susceptible cheats and resistant cooperators (e.g. vi), only cooperators gain resistance (e.g. vii), and both cooperators and cheats gain resistance (e.g. viii). These outcomes are shown in figure S4, with explicit examples of typical simulation dynamics for each scenario detailed below in figure S5.


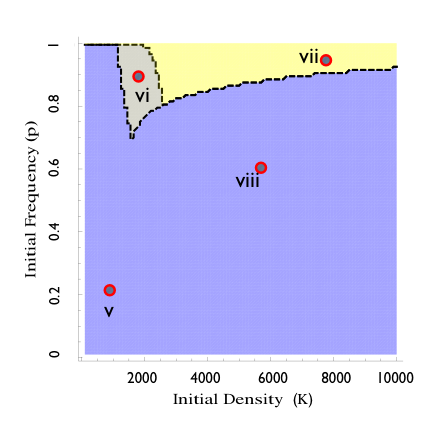


Figure S4: Redrawing figure S1 for a high cost of cooperation. Cheats are reaching fixation across the majority of the parameter space. The yellow area is where only cooperators gain resistance, hence only *C_R_* are present at the end of the simulation (e.g. vi). The grey area is where susceptible cheats (*D_S_*) are persisting with resistant cooperators (e.g. vi). The blue area is where cheats reach fixation - whether it be due to *D_S_* outcompeting *C_S_* (e.g. v) or *D_R_* outcompeting *C_R_* (e.g. viii). Representative time-series plots for each of the scenarios v-viii are pictured in figure S5. Parameters: *r* = 1, *μ* = 0.01, *y* = 10, *e* = 0.0001, *φ* = 0.2.

Figure S5: Examples of population dynamics from specified points in figure S4. Within each panel, the top graph is a time series showing numbers of *C_s_* (green, dashed), *D_s_* (red, dashed), *C_R_* (dark green), *D_R_* (dark red), and virus (purple) through arbitrary units of time. Note: viral numbers are scaled down (/15) such that the results can be easily presented on the same axes. The bottom graphs in each panel correspond to those above, showing the total proportion of cooperators (*C_S_* + *C_R_*, green) and cheats (*D_S_* + *D_R_,* red) in the population. v) Neither *C* or *D* gain resistance; vi) Coexistence of *D_S_* and *C_R_*; vii) Only *C* gain resistance; viii) Both *C* and *D* gain resistance. Parameters: *r* = 1, *K* = variable, *μ* = 0.01, *y* = 10, *e* = 0.0001, *φ* = 0.2, *b* = 0.5, *c* =.0.49.

In these simulations we demonstrate, with explicit dynamics, how cooperation can be maintained in an unstructured population. The neutral model can be used in a broader context than cooperation and cheating. Given the symmetry in the model, the advantage lies with the more abundant i.e. positive frequency dependence. Once we introduce cooperation and defection, the symmetry is broken, and cooperators must be initially more common than cheats to be maintained by hitchhiking with the resistance mutation. However, we argue that cheats are likely to be invading from rare in natural populations (see main manuscript) and that this model represents a biologically plausible mechanism which may facilitate the maintenance of cooperation. Stochastic simulations of these equations generate qualitatively very similar results.

A limitation of these simulation results is that they do not allow for further mutation of the resistant strains, meaning that they can only explain the maintenance of cooperation in the short term. Further stochastic simulations have been conducted to explore the effect of an ongoing evolutionary arms race between host and parasite, indicating that this same mechanism can enable the frequency dependent maintenance of cooperation in the longer term (Quigley *et al* submitted).

**Supporting figures**

**Figure S6**

**Short term (48 hours) growth rates of cheat (a) and cooperator (b).** Cheats and wild-type were grown as monocultures (100% starting frequency) and in the presence of each other, where cheat was mixed with the cooperator at starting frequencies of 50% of each, and at 0.01% cheat with 99.99% wild-type. Growth rates were compared by calculating the Malthusian parameters where  *m* = *ln*(*N*_f_/*N*_0_). *N*_0_ is the starting density and *N*_f_ is the final density. Error bars are ± 1 standard error of the mean.

Figure S7

**Figure S7**

**
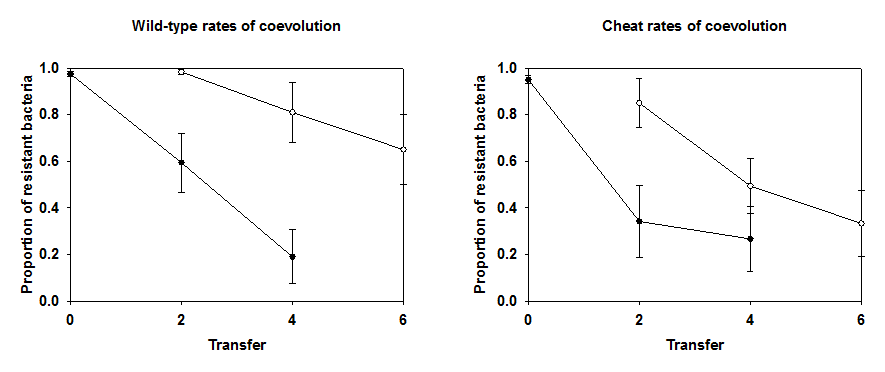
**

**Comparison of rates of coevolution between pure cultures of wild-type and cheat.**  The proportion of 20 bacterial colonies from transfers 2 (black circles) and 4 (white circles) resistant to phages from two transfers previous, contemporary phages, and to phages from two transfers in the future. Error bars are ± 1 standard error of the mean. Negative slopes show that the resistance of the bacteria is lower to phages from later time points, and hence phage infectivity is increasing. The lines in both the wild-type and cheat populations from transfer 4 are above the lines from transfer 2 which shows that bacterial resistance is also increasing through time. For example, the data points at transfer 2 on the x-axis are the resistance of the bacteria from two different time points (the two separate lines) to the phage from transfer 2. The bacteria from transfer 4 is more resistant than the bacteria from transfer 2 to the phage from transfer 2, because the bacteria has evolved resistance.
